# Association between serum 25-hydroxyvitamin D, nutritional status, and inflammatory markers in sepsis patients with and without sarcopenia

**DOI:** 10.3389/fmed.2026.1816016

**Published:** 2026-04-13

**Authors:** Hong Zheng, Chaoyong Bei, Zhongcheng Mo, Bing Wei

**Affiliations:** 1 Department of Emergency Medicine, The First Affiliated Hospital of Guilin Medical University, Guilin, Guangxi Zhuang Autonomous Region, China; 2 Guangxi Key Laboratory of Diabetic Systems Medicine, Guilin Medical University, Guilin, Guangxi Zhuang Autonomous Region, China; 3 Department of Geriatrics, The First Affiliated Hospital of Guilin Medical University, Guilin, Guangxi Zhuang Autonomous Region, China; 4 Department of Trauma and Extremity Orthopedics, The First Affiliated Hospital of Guilin Medical University, Guilin, Guangxi Zhuang Autonomous Region, China; 5 School of Medical Engineering Technology, Hunan Institute of Engineering, Xiangtan, Hunan, China

**Keywords:** cut-off values, inflammation, nutritional status, sarcopenia, sepsis, vitamin D

## Abstract

**Background:**

Sepsis, a critical infectious syndrome, frequently presents with sarcopenia and is typified by an inflammatory cascade and vitamin D (VD) deficiency. The interrelation between these conditions remains to be fully elucidated. VD, known for its immunomodulatory properties, is suspected to play a role in the pathogenesis of both sepsis and sarcopenia. The significance of serum VD levels in diagnosing sepsis and sarcopenia is well-recognized. This study was designed to scrutinize the relationships among VD, nutritional status, and inflammatory profiles in individuals with sepsis and concurrent sarcopenia, and to explore potential VD cut-off values.

**Methods:**

The study cohort comprised two principal groups: a sepsis group and a control group. The sepsis group was further bifurcated into those with and without sarcopenia. The study enrolled 100 patients diagnosed with sepsis from February 2022 to February 2023, and 92 healthy controls. Of the sepsis patients, 46 had comorbid sarcopenia. Serum 25(OH)D levels, along with nutritional and inflammatory biomarkers, were assessed, and receiver operating characteristic (ROC) curve analysis was performed to determine the optimal cut-off values.

**Results:**

Notable disparities in 25(OH)D levels and markers of nutrition and inflammation were observed between the sepsis and control groups, as well as between the subgroups with and without sarcopenia. The optimal cut-off values of serum 25(OH)D derived from ROC analysis were 22.55 ng/mL for sepsis and 14.31 ng/mL for sepsis with sarcopenia.

**Conclusion:**

Serum 25(OH)D levels were significantly associated with nutritional status and inflammatory responses in patients with sepsis, particularly in those with sarcopenia, and may serve as a potential biomarker reflecting these clinical features.

## Highlights

- This investigation provides a nuanced understanding of the relationship between VD deficiency, sarcopenia, and sepsis, accentuating the significance of VD in immune modulation.
- It reveals pronounced differences in serum 25(OH)D levels and related inflammatory and nutritional indicators among sepsis patients, with and without sarcopenia, in contrast to control subjects.
- The study delineates superior VD threshold criteria for sepsis and its concomitant sarcopenia, highlighting the clinical relevance of VD measurements in the diagnosis and morbidity forecasting for these patient groups.

## 1 Introduction

Sepsis is a life-threatening organ dysfunction caused by a dysregulated host response to infection, characterized by complex immune dysfunction and inflammatory responses (1, 2). In addition to systemic inflammation, sepsis is also accompanied by profound metabolic disturbances and immune imbalance, both of which may influence clinical outcomes (1). The pathogenesis of this condition is multifaceted and not yet fully understood. In recent years, the potential role of vitamin D (VD) in the pathogenesis of sepsis has garnered significant interest. Evidence suggests that VD exerts its influence not only on immune cells but also on a variety of cellular types pivotal to bone and mineral metabolism (3, 4). The regulatory function of VD in modulating immune responses and combating infection is well-documented, and its interplay with inflammatory pathways is a subject of extensive research (5–7). Although the precise mechanisms remain incompletely understood, growing evidence suggests that vitamin D may influence sepsis-related immune and inflammatory responses through modulation of innate and adaptive immunity, cytokine production, and antimicrobial defense. In observational studies, low serum 25-hydroxyvitamin D (25[OH]D) levels have been associated with a higher risk and greater severity of sepsis, as well as with increased inflammatory burden (8–10).

Since the 1980s, a high prevalence of sarcopenia—characterized by progressive muscle mass and function decline—has been reported among the elderly (11). Sarcopenia is intricately linked to age-related physiological alterations, malnutrition, chronic illnesses, inflammation, and lifestyle factors (12). Emerging research suggests that VD deficiency may be associated with the development and progression of sarcopenia, with low VD levels implicated not only in bone metabolism but also in skeletal muscle physiology. VD may affect muscle health through multiple pathways, including muscle protein synthesis, mitochondrial function, calcium homeostasis, and the modulation of inflammatory signaling. Furthermore, its regulatory impact on immune function and inflammatory responses may also contribute to the development of sarcopen (13).

Recent investigations have suggested a link between sarcopenia and the onset and severity of sepsis (14, 15). Patients with sepsis often exhibit immune dysregulation, pronounced inflammatory responses, catabolic stress, reduced mobility, and nutritional insufficiency, all of which may contribute to accelerated muscle loss and the development of sarcopenia (16, 17). Conversely, those with sarcopenia are at an increased risk of malnutrition and immune dysfunction at sepsis onset, potentially related to VD’s role in immune modulation and nutrient metabolism (18). Collectively, these findings suggest that VD deficiency may be associated with both sepsis and sarcopenia, possibly through shared pathways involving immune dysregulation, inflammation, and altered muscle metabolism (19, 20). While the exact role of VD in sepsis and sarcopenia requires further investigation, current evidence supports a potential link between VD status, immune responses, and nutritional/metabolic disturbances in these conditions (21).

The potential clinical relevance of VD deficiency in sepsis and sarcopenia has attracted increasing attention (22, 23). Systematic reviews and meta-analyses have supported an association between VD deficiency and both sepsis and sarcopenia, although these findings are largely based on observational evidence (24, 25). Although several studies have explored the potential role of VD as a biomarker, data on clinically relevant 25(OH)D thresholds in patients with sepsis, particularly those with sarcopenia, remain limited (26–30). The current study aimed to address this gap by exploring 25(OH)D cut-off values with potential discriminatory value for sepsis and sepsis with sarcopenia, particularly in a Chinese patient population where relevant data remain scarce.

Given the reported associations of VD with nutritional status and inflammation, serum 25(OH)D may have potential value as a biomarker reflecting nutritional and inflammatory status in patients with sepsis and comorbid sarcopenia. Therefore, this study evaluated VD levels in relation to nutritional status and inflammatory markers and further explored their potential discriminatory value by estimating 25(OH)D cut-off values.

## 2 Materials and methods

### 2.1 Ethics statement

The study was dutifully approved by the Ethics Committee of the Affiliated Hospital of Guilin Medical University (2022QTLL-45), with the clinical trial registered under the identifier ChiCTR2200060595.^1^ In strict adherence to the Helsinki Declaration, written informed consent was obtained from all participants prior to study enrollment. All personnel involved in the study underwent rigorous training to ensure standardization of laboratory techniques and procedural uniformity.

### 2.2 Patients

This was a single-center cross-sectional study conducted in the Department of Emergency Medicine of the Affiliated Hospital of Guilin Medical University. A total of 100 patients with sepsis were enrolled, including 46 patients with concomitant sarcopenia. Participants were consecutively recruited between February 2022 and February 2023 before the initiation of treatment. Eligible sepsis patients were consecutively screened and recruited in the Department of Emergency Medicine during the study period, and healthy controls were recruited during the same period as the comparative group. The sample size was determined by the number of eligible participants who were consecutively recruited during the study period.

The inclusion criteria for the sepsis group were consecutive patients admitted during the study period who met the Sepsis-3 diagnostic criteria and had not initiated treatment at the time of enrollment. The exclusion criteria were incomplete clinical or laboratory data and inability to provide informed consent. Healthy controls were recruited during the same period and had no evidence of acute infection or sepsis at enrollment. The sepsis diagnosis was ascertained through a synthesis of clinical assessments, biochemical analyses, bacteriological examinations, and histological evaluations, in strict accordance with the Sepsis-3 criteria established in 2016.

For the detection of sarcopenia in the sepsis cohort, a multifaceted approach was employed, integrating clinical presentations, thorough physical examinations, diagnostic imaging, and the authoritative guidelines provided by the Asian Working Group on Sarcopenia (AWGS) as outlined in their 2019 Consensus on the Diagnosis and Treatment of Sarcopenia. Specifically, the diagnosis was based on objective assessments of muscle mass, muscle strength, and physical performance in accordance with the AWGS 2019 criteria. Concurrently, a control group comprising 92 healthy volunteers was assembled to serve as a comparative reference in the study.

### 2.3 Measurements

Sarcopenia-related assessments included the evaluation of muscle mass, muscle strength, and physical performance according to the AWGS 2019 criteria. Nutritional status was assessed using the Nutritional Risk Screening 2002 (NRS-2002), the Scored Patient-Generated Subjective Global Assessment (PG-SGA), and Body Mass Index (BMI). These tools were used to comprehensively evaluate nutritional risk and nutritional status in the study participants. The NRS-2002 was used to screen for nutritional risk, and a score of ≥3 was considered indicative of nutritional risk. The PG-SGA was used to evaluate nutritional status, and a score of ≥4 was considered indicative of malnutrition. Anthropometric measurements, height and weight, were recorded with precision to the nearest 0.1 cm and 0.1 kg, respectively, using standardized scales while participants were barefoot and unclothed save for light attire. BMI was calculated using the formula weight in kilograms divided by the square of height in meters. The classification of BMI was as follows: underweight (BMI < 18.5 kg/m^2^), normal weight (18.5 kg/m^2^ ≤ BMI < 25 kg/m^2^), overweight (25 kg/m^2^ ≤ BMI < 30 kg/m^2^), and obese (BMI ≥ 30 kg/m^2^), with underweight being indicative of a nutritional deficiency.

Furthermore, we utilized the Sequential Organ Failure Score (SOFA), Acute Physiology and Chronic Health Evaluation II (APACHE II), and the Nutritional Risk Score for the Critically Ill Patient (NUTRIC), along with metrics for the incidence of septic shock and the 28-day survival rate, to gauge the inflammatory response and the severity of sepsis. The SOFA score quantified the extent of organ dysfunction, with higher scores correlating with increased infection severity and organ failure. The APACHE II score evaluated the overall disease severity, with higher scores foretelling graver conditions and a more adverse prognosis. The NUTRIC score assessed the nutritional risk and concurrent disease severity in critically ill patients, with higher scores reflecting greater nutritional risk and more intense disease manifestations.

Laboratory data encompassed measurements of Total Protein (TP), Albumin (ALB), C-reactive protein (CRP), Procalcitonin (PCT), Interleukin-6 (IL-6), and serum 25-hydroxyvitamin D (25[OH]D). CRP and IL-6 were quantified using the Enzyme-Linked Immunosorbent Assay (ELISA), Serum 25(OH)D levels were measured by radioimmunoassay using a DiaSorin analyzer (DiaSorin, Stillwater, MN, USA), PCT levels were determined by chemiluminescence, and TP and ALB were measured using standard biochemical methods. Data collection was uniformly conducted in the morning following an 8- to 12-h fasting period, with each participant assessed by the same researcher to ensure consistency. Blood samples were systematically collected in 5 mL anticoagulant-free sodium heparin tubes and anticoagulant-free tubes. A qualified physician was responsible for the routine collection of medical history and the assessment of nutritional and inflammatory biomarkers.

### 2.4 Statistical analysis

Statistical analysis was performed utilizing SPSS version 26.0 (IBM Corp., Armonk, NY, USA). We employed descriptive statistical measures to delineate the demographic and clinical characteristics of the study sample. Quantitative variables were summarized as means and standard deviations, and categorical variables were presented as numbers and percentages. Comparative analyses of indicators between groups were executed using Student’s *t*-test for variables exhibiting normal distribution, while the Mann–Whitney *U* test was applied for variables that did not adhere to normal distribution. Categorical variables were compared using the chi-square test or Fisher’s exact test, as appropriate. The assessment of correlations between various indicators was conducted through linear regression for continuous variables and binary logistic regression analysis for categorical outcomes. Statistical significance was set at the *p*-value threshold of less than 0.05.

Graphical representations of the data were crafted using GraphPad Prism software, version 8.0.2 (GraphPad Software, San Diego, CA, USA). A receiver operating characteristic (ROC) analysis was implemented to evaluate the diagnostic sensitivity and specificity of serum 25-hydroxyvitamin D (25[OH]D) levels in distinguishing sepsis and sepsis with sarcopenia. This analysis also served to identify the optimal cut-off value for vitamin D levels and to explore their potential discriminatory value.

## 3 Results

**Figure 1:**
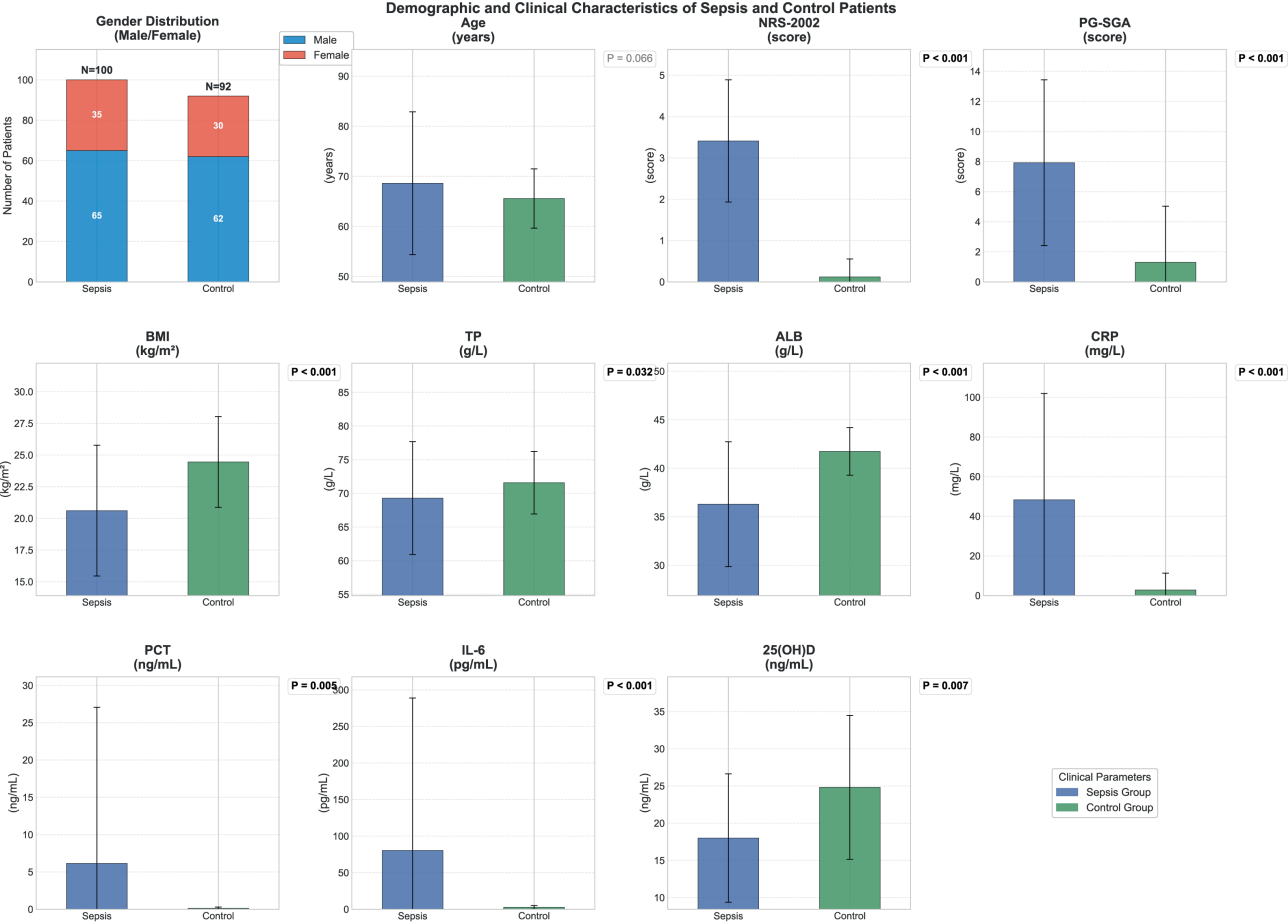
Demographic and clinical characteristics of sepsis and control patients.

Upon examination of the data presented in Figure 1. The detailed underlying data are provided in Table 1, several notable clinical distinctions emerged between sepsis patients and the control group. The average age of individuals in the sepsis cohort, at 68.630 ± 14.263 years, was marginally elevated in comparison to the control group’s average of 65.577 ± 5.916 years; however, this variance did not reach a level of statistical significance (*p* = 0.066).

**Table 1:** Demographic and clinical characteristics of sepsis and control patients.

Characteristic	Sepsis	Control	*P*
*N* (male/female)	100 (65/35)	92 (62/30)	–
Age (years)	68.630 ± 14.263	65.577 ± 5.916	0.066
NRS-2002	3.410 ± 1.478	0.120 ± 0.435	<0.001
PG-SGA	7.920 ± 5.513	1.308 ± 3.723	<0.001
BMI (kg/m^2^)	20.614 ± 5.161	24.458 ± 3.585	<0.001
TP (g/L)	69.297 ± 8.382	71.589 ± 4.638	0.032
ALB (g/L)	36.302 ± 6.429	41.741 ± 2.456	<0.001
CRP (mg/L)	48.306 ± 53.582	2.834 ± 8.474	<0.001
PCT (ng/mL)	6.152 ± 20.928	0.119 ± 0.175	0.005
IL-6 (pg/mL)	80.358 ± 208.478	2.662 ± 2.392	<0.001
Serum 25(OH)D (ng/mL)	18.002 ± 8.629	24.822 ± 9.663	0.007

Data are mean values ± standard deviation (SD). NRS-2002: Nutrition Risk Screening 2002; PG-SGA score: Patient-Generated Subjective Global Assessment score; BMI: Body Mass Index; TP: Total Protein; ALB: Albumin; CRP: C-reactive protein; PCT: Procalcitonin; IL-6: Interleukin 6. *p*-values were calculated using Student’s *t*-test for normally distributed variables or Mann–Whitney *U* test for those not normally distributed. Differences were considered significant at *p* < 0.05.

Significant disparities were observed in the NRS-2002 and PG-SGA scores, with the sepsis group consistently demonstrating higher scores: 3.410 ± 1.478 versus 0.120 ± 0.435 (*p* < 0.001) for NRS-2002, and 7.920 ± 5.513 versus 1.308 ± 3.723 (*p* < 0.001) for PG-SGA, respectively. Additionally, the body mass index (BMI) of the sepsis group was markedly reduced compared to the control group, with respective averages of 20.614 ± 5.161 and 24.458 ± 3.585 (*p* < 0.001), indicating a pronounced malnutrition profile.

Biochemical indices further delineated the divergence between the two groups. The sepsis group exhibited significantly diminished levels of TP and ALB, with averages of 69.297 ± 8.382 and 36.302 ± 6.429, contrasting with the control group’s 71.589 ± 4.638 and 41.741 ± 2.456 (*p* = 0.032 for TP, *p* < 0.001 for ALB). Conversely, the sepsis group displayed elevated levels of CRP, PCT, and IL-6: 48.306 ± 53.582 versus 2.834 ± 8.474 (*p* < 0.001) for CRP, 6.152 ± 20.928 versus 0.119 ± 0.175 (*p* = 0.005) for PCT, and 80.358 ± 208.478 versus 2.662 ± 2.392 (*p* < 0.001) for IL-6. Furthermore, serum 25(OH)D levels were significantly depressed in the sepsis group, with an average of 18.002 ± 8.629 compared to the control group’s 24.822 ± 9.663 (*p* = 0.007).

In essence, the clinical and biochemical profiles of sepsis patients reveal a starkly malnourished and inflammatory phenotype, which may be intricately connected to the etiology and clinical progression of sepsis.

**Figure 2:**
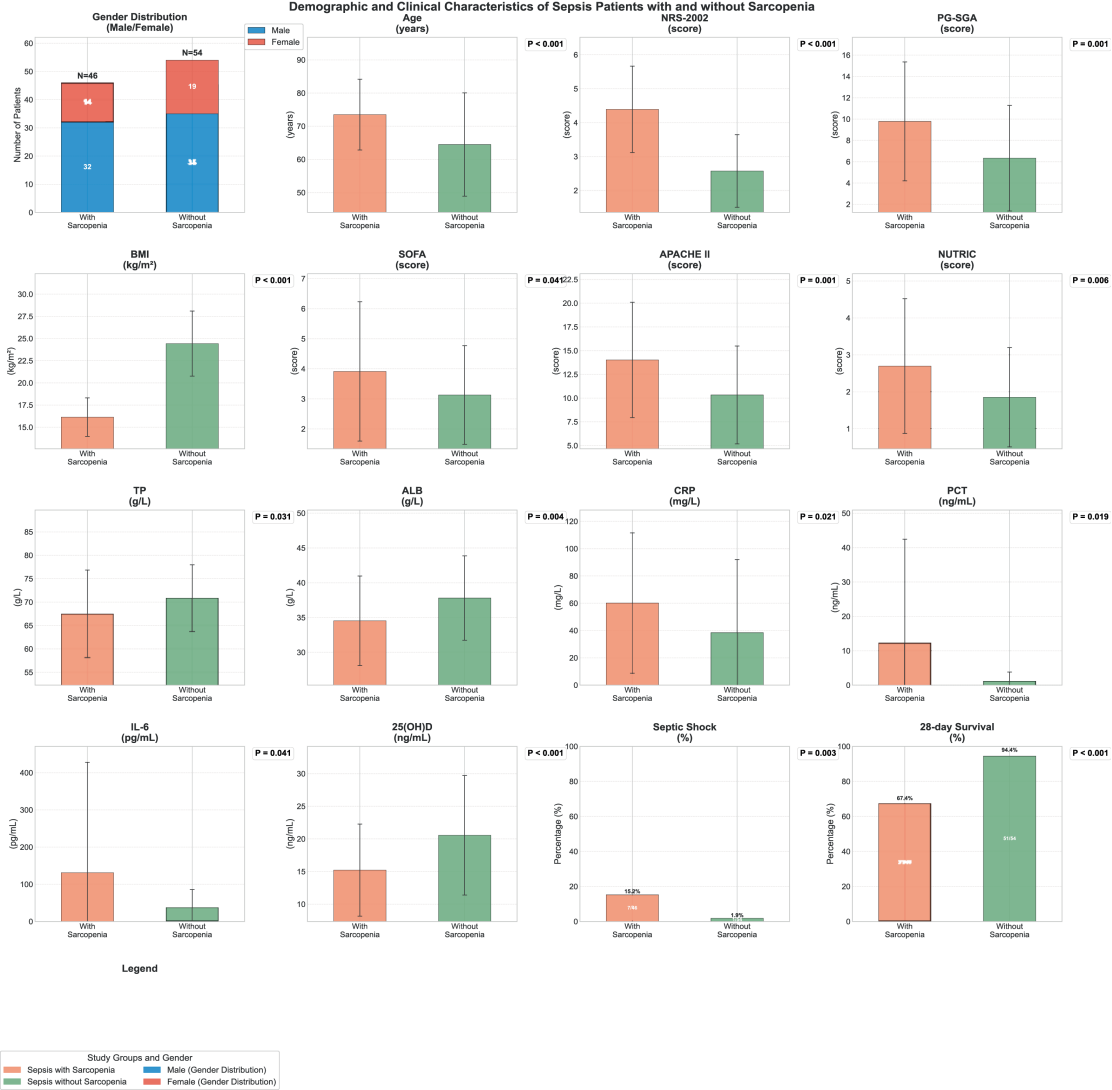
Demographic and clinical characteristics of patients with sepsis.

The data were analyzed to discern the impact of sarcopenia on sepsis patients, with the sepsis cohort stratified into those with and without sarcopenia for a comparative study. As delineated in Figure 2. The detailed underlying data are provided in Table 2, substantial disparities were noted between these two subgroups within the sepsis population.

**Table 2:** Demographic and clinical characteristics of patients with sepsis.

Characteristic	Sepsis with sarcopenia	Sepsis without sarcopenia	*p*
*N* (male/female)	46 (32/14)	54 (35/19)	–
Age (years)	73.500 ± 10.672	64.481 ± 15.584	<0.001
NRS-2002	4.391 ± 1.273	2.574 ± 1.070	<0.001
PG-SGA	9.783 ± 5.581	6.333 ± 4.952	0.001
BMI (kg/m^2^)	16.139 ± 2.167	24.426 ± 3.668	<0.001
SOFA	3.913 ± 2.317	3.130 ± 1.641	0.041
APACHE II	14.022 ± 6.072	10.333 ± 5.152	0.001
NUTRIC	2.696 ± 1.824	1.852 ± 1.345	0.006
The incidence rate of septic shock (%)	15.217 (7/46)	1.852 (1/54)	0.003
28-day survival rate (%)	67.391 (31/46)	94.444 (51/54)	<0.001
TP (g/L)	67.462 ± 9.383	70.860 ± 7.114	0.031
ALB (g/L)	34.538 ± 6.432	37.804 ± 6.062	0.004
CRP (mg/L)	60.147 ± 51.407	38.439 ± 53.568	0.021
PCT (ng/mL)	12.284 ± 30.157	1.157 ± 2.654	0.019
IL-6 (pg/mL)	131.084 ± 296.879	37.289 ± 48.554	0.041
Serum 25(OH)D (ng/mL)	15.222 ± 7.054	20.559 ± 9.159	<0.001

Data are presented as mean ± standard deviation (SD). NRS-2002, Nutrition Risk Screening 2002; PG-SGA score, Patient-Generated Subjective Global Assessment score; BMI, Body Mass Index; SOFA, Sequential Organ Failure Assessment; APACHE II, Acute Physiology and Chronic Health Evaluation II; NUTRIC, Nutrition Risk in Critically Ill; TP, Total Protein; ALB, Albumin; CRP, C-reactive protein; PCT, Procalcitonin; IL-6, Interleukin 6. Statistical significance was determined using Student’s *t*-test for normally distributed variables or Mann–Whitney *U* test for non-normally distributed variables, with differences considered significant at *p* < 0.05.

The mean age of the sepsis group complicated by sarcopenia (73.500 ± 10.672 years) was notably elevated compared to the sepsis group without sarcopenia (64.481 ± 15.584 years), with this difference attaining statistical significance (*p* < 0.001). The sepsis group with sarcopenia also exhibited markedly higher scores in the NRS-2002 and PG-SGA assessments, with respective values of 4.391 ± 1.273 versus 2.574 ± 1.070 (*p* < 0.001) and 9.783 ± 5.581 versus 6.333 ± 4.952 (*p* = 0.001). Furthermore, this group demonstrated a significantly reduced BMI (16.139 ± 2.167 vs. 24.426 ± 3.668, *p* < 0.001), underscoring a pronounced malnutrition phenotype.

Clinical scoring systems indicated a trend towards higher severity in the sepsis group with sarcopenia, as evidenced by the SOFA score (3.913 ± 2.317 vs. 3.130 ± 1.641, *p* = 0.041) and APACHE II score (14.022 ± 6.072 vs. 10.333 ± 5.152, *p* = 0.001). Similarly, the NUTRIC score was modestly elevated in the sepsis group with sarcopenia (2.696 ± 1.824 vs. 1.852 ± 1.345, *p* = 0.006). Clinical outcome parameters included the incidence of septic shock and the 28-day survival rate. The rate of septic shock was significantly more prevalent in the sepsis group with sarcopenia (15.217% vs. 1.852%, *p* = 0.003), while the 28-day survival rate was considerably lower in this group (67.391% vs. 94.444%, *p* < 0.001), indicating a poorer prognosis.

Biochemical parameters revealed that levels of TP and ALB were significantly lower, whereas CRP, PCT, and IL-6 were significantly higher in the sepsis group with sarcopenia compared to the non-sarcopenia group, with respective values of 67.462 ± 9.383 vs. 70.860 ± 7.114 (*p* = 0.031), 34.538 ± 6.432 vs. 37.804 ± 6.062 (*p* = 0.004), 60.147 ± 51.407 vs. 38.439 ± 53.568 (*p* = 0.021), 12.284 ± 30.157 vs. 1.157 ± 2.654 (*p* = 0.019), and 131.084 ± 296.879 vs. 37.289 ± 48.554 (*p* = 0.041). Additionally, serum 25(OH)D levels were significantly reduced in the sepsis group with sarcopenia (15.222 ± 7.054 vs. 20.559 ± 9.159, *p* < 0.001).

In summary, sepsis patients with concomitant sarcopenia displayed significant malnutrition, a robust inflammatory response, and a higher incidence of septic shock coupled with a diminished survival rate. These clinical features and biochemical indices suggest a strong correlation between sarcopenia and adverse outcomes in sepsis.

**Figure 3:**
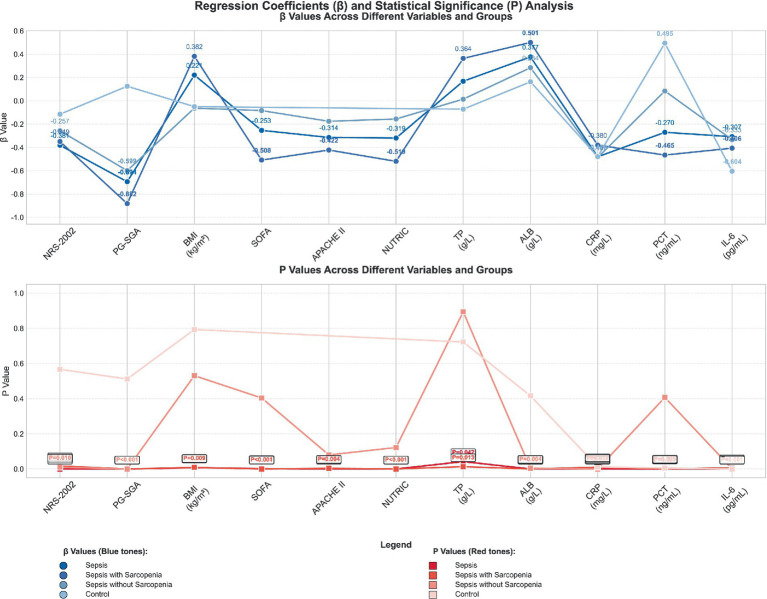
Regression analysis between serum 25(OH)D and nutritional, inflammatory indicators for total sepsis, sepsis with sarcopenia, sepsis without sarcopenia and control groups.

The regression analyses elucidating the relationships between serum 25(OH)D levels and various indices are show in Figure 3, The detailed underlying data are provided in Table 3. This table encapsulates the findings from regression models assessing the association of serum 25(OH)D with nutritional and inflammatory markers across different groups: sepsis patients, those with sepsis and sarcopenia, those with sepsis without sarcopenia, and a control cohort. In the sepsis group, serum 25(OH)D exhibited a significant negative correlation with the NRS-2002, PG-SGA, SOFA, APACHE II, and NNUTRIC scores. Conversely, it demonstrated a positive correlation with BMI, TP, and ALB.

**Table 3:** Regression analysis between serum 25(OH)D and nutritional, inflammatory indicators for total sepsis, sepsis with sarcopenia, sepsis without sarcopenia and control groups.

Characteristic	Sepsis		Sepsis with sarcopenia		Sepsis without sarcopenia		Control	
	*β*	*p*	*β*	*p*	*β*	*p*	*β*	*p*
NRS-2002	−0.381	<0.001	−0.349	0.017	−0.257	0.010	−0.115	0.567
PG-SGA	−0.694	<0.001	−0.882	<0.001	−0.599	<0.001	0.125	0.512
BMI (kg/m^2^)	0.221	0.007	0.382	0.009	−0.063	0.531	−0.050	0.793
SOFA	−0.253	0.002	−0.508	<0.001	−0.084	0.404	–	–
APACHE II	−0.314	<0.001	−0.422	0.004	−0.176	0.080	–	–
NUTRIC	−0.319	<0.001	−0.519	<0.001	−0.156	0.122	–	–
TP (g/L)	0.168	0.042	0.364	0.013	0.014	0.894	−0.072	0.722
ALB (g/L)	0.377	<0.001	0.501	<0.001	0.284	0.004	0.163	0.416
CRP (mg/L)	−0.480	<0.001	−0.380	0.010	−0.486	<0.001	−0.479	0.012
PCT (ng/mL)	−0.270	0.001	−0.465	0.001	0.084	0.407	0.495	0.005
IL-6 (pg/mL)	−0.307	<0.001	−0.406	0.006	−0.333	0.001	−0.604	<0.001

NRS-2002, Nutrition Risk Screening 2002; PG-SGA score, Patient-Generated Subjective Global Assessment score; BMI, Body Mass Index; SOFA, Sequential Organ Failure Assessment; APACHE II, Acute Physiology and Chronic Health Evaluation II; NUTRIC, Nutrition Risk in Critically Ill; TP, Total Protein; ALB, Albumin; CRP, C-reactive protein; PCT, Procalcitonin; IL-6, Interleukin 6.

Among the sepsis patients with sarcopenia, the negative correlation between serum 25(OH)D and inflammatory markers was more pronounced, with correlation coefficients of *β* = −0.380 for CRP (*p* = 0.010), *β* = −0.465 for PCT (*p* = 0.001), and *β* = −0.406 for IL-6 (*p* = 0.006), indicating a stronger relationship with inflammatory burden. In contrast, in the sepsis group without sarcopenia, serum 25(OH)D was also negatively correlated with CRP (*β* = −0.486, *p* < 0.001) and IL-6 (*β* = −0.333, *p* = 0.001), whereas the association with PCT was not statistically significant (*β* = 0.084, *p* = 0.407). In the control group, serum 25(OH)D levels were negatively associated with the NRS-2002, PG-SGA, CRP, and IL-6, while a positive association was observed with BMI, TP, ALB, and PCT.

These regression analyses underscore a discernible link between serum 25(OH)D levels and the nutritional and inflammatory profiles of sepsis patients, particularly within the subgroup presenting with both sepsis and sarcopenia. The findings hint at the potential role of vitamin D in modulating the inflammatory and nutritional dynamics in critical illness.

**Figure 4:**
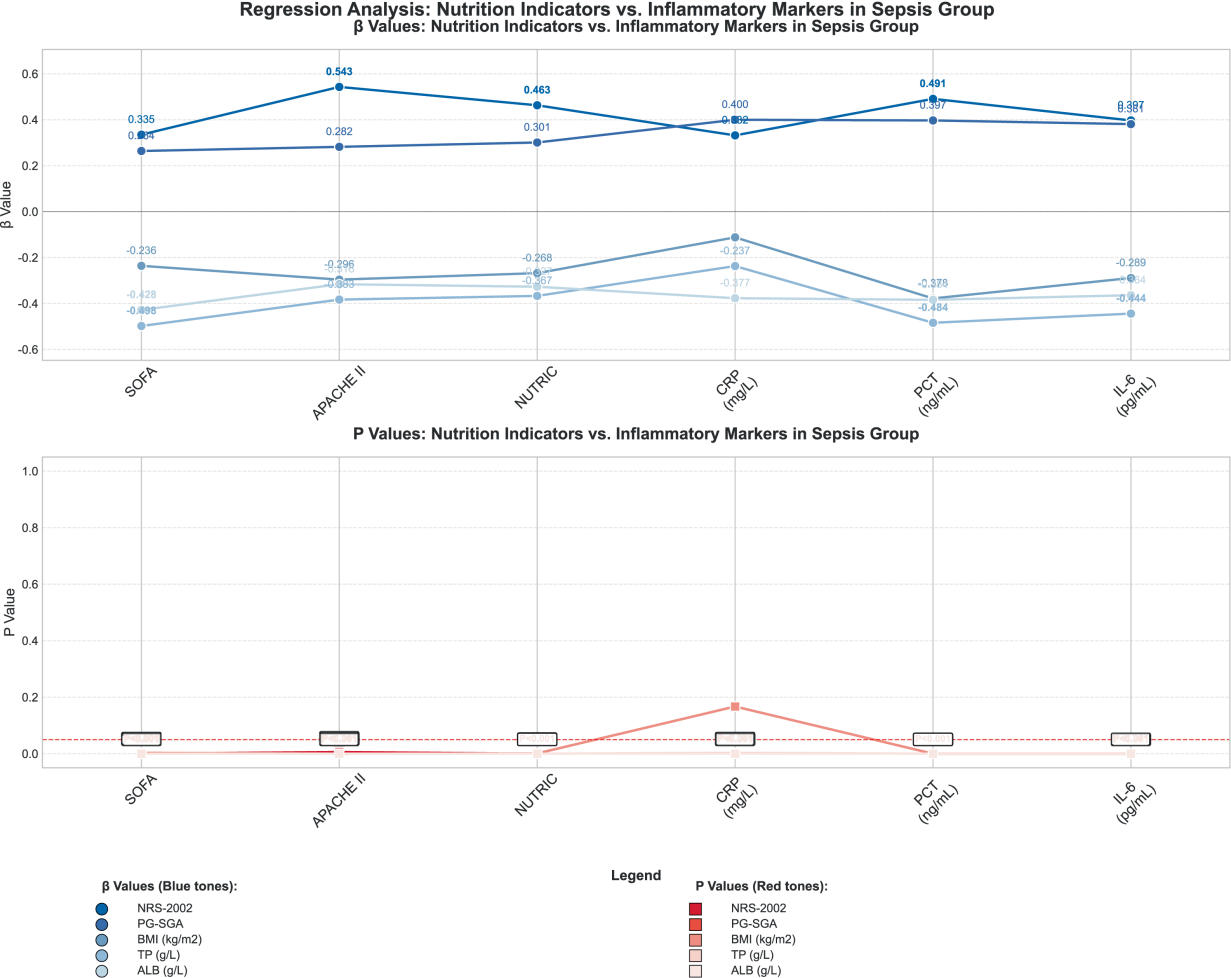
Regression analysis between nutrition indicators and inflammatory markers in sepsis group.

**Figure 5:**
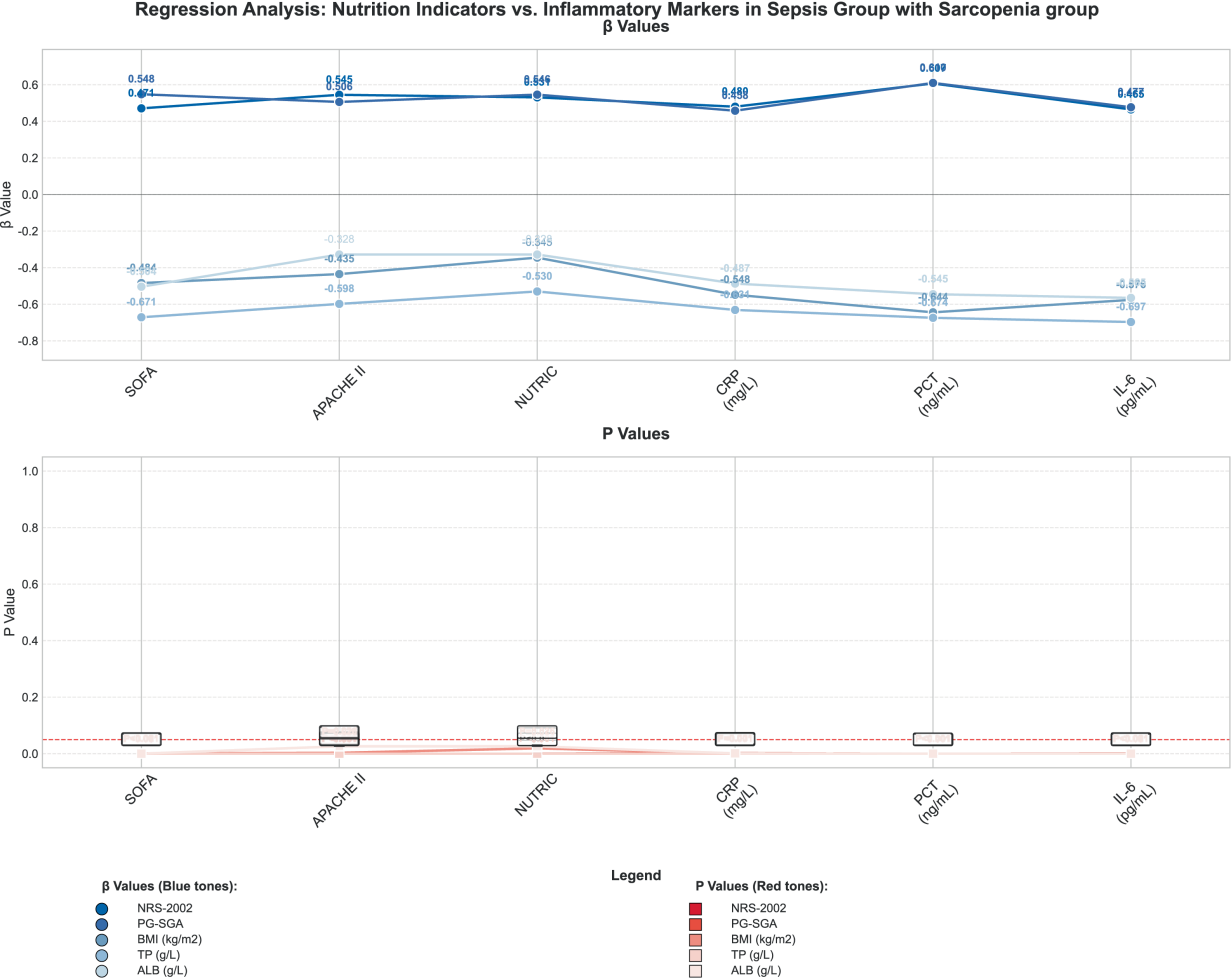
Regression analysis between nutrition indicators and inflammatory markers in sepsis with sarcopenia group.

The regression analyses, with the key results visualized in Figures 4 and 5 and the full statistical details provided in Tables 4 and 5, yield several key insights into the interplay between nutritional and inflammatory markers within the sepsis population. In the sepsis group, a correlation was identified between nutritional indicators—encompassing the NRS-2002, PG-SGA, BMI, TP, and ALB—and inflammatory markers, which included the SOFA, APACHE II, NUTRIC, CRP, PCT, and IL-6.

**Table 4:** Regression analysis between nutrition indicators and inflammatory markers in sepsis group.

Characteristic	NRS-2002		PG-SGA		BMI (kg/m^2^)		TP (g/L)		ALB (g/L)	
	*β*	*p*	*β*	*p*	*β*	*p*	*β*	*p*	*β*	*p*
SOFA	0.335	<0.001	0.264	0.001	−0.236	0.003	−0.498	<0.001	−0.428	<0.001
APACHE II	0.543	0.006	0.282	<0.001	−0.296	<0.001	−0.383	<0.001	−0.316	<0.001
NUTRIC	0.463	<0.001	0.301	<0.001	−0.268	0.001	−0.367	<0.001	−0.327	<0.001
CRP (mg/L)	0.332	<0.001	0.400	<0.001	−0.112	0.167	−0.237	0.003	−0.377	<0.001
PCT (ng/mL)	0.491	<0.001	0.397	<0.001	−0.378	<0.001	−0.484	<0.001	−0.384	<0.001
IL-6 (pg/mL)	0.397	<0.001	0.381	<0.001	−0.289	<0.001	−0.444	<0.001	−0.364	<0.001

NRS-2002, Nutrition Risk Screening 2002; PG-SGA score, Patient-Generated Subjective Global Assessment score; BMI, Body Mass Index; SOFA, Sequential Organ Failure Assessment; APACHE II, Acute Physiology and Chronic Health Evaluation II; NUTRIC, Nutrition Risk in Critically Ill; TP, Total Protein; ALB, Albumin; CRP, C-reactive protein; PCT, Procalcitonin; IL-6, Interleukin 6.

**Table 5:** Regression analysis between nutrition indicators and inflammatory markers in sepsis with sarcopenia group.

Characteristic	NRS-2002		PG-SGA		BMI (kg/m^2^)		TP (g/L)		ALB (g/L)	
	*β*	*p*	*β*	*p*	*β*	*p*	*β*	*p*	*β*	*p*
SOFA	0.471	0.001	0.548	<0.001	−0.484	0.001	−0.671	<0.001	−0.504	<0.001
APACHE II	0.545	<0.001	0.506	<0.001	−0.435	0.003	−0.598	<0.001	−0.328	0.026
NUTRIC	0.531	<0.001	0.546	<0.001	−0.345	0.019	−0.530	<0.001	−0.328	0.026
CRP (mg/L)	0.480	0.001	0.458	0.002	−0.548	<0.001	−0.631	<0.001	−0.487	0.001
PCT (ng/mL)	0.607	<0.001	0.610	<0.001	−0.644	<0.001	−0.674	<0.001	−0.545	<0.001
IL-6 (pg/mL)	0.465	0.001	0.477	0.001	−0.576	<0.001	−0.697	<0.001	−0.565	<0.001

NRS-2002, Nutrition Risk Screening 2002; PG-SGA score, Patient-Generated Subjective Global Assessment score; BMI, Body Mass Index; SOFA, Sequential Organ Failure Assessment; APACHE II, Acute Physiology and Chronic Health Evaluation II; NUTRIC, Nutrition Risk in Critically Ill; TP, Total Protein; ALB, Albumin; CRP, C-reactive protein; PCT, Procalcitonin; IL-6, Interleukin 6.

Notably, the NRS-2002, PG-SGA, and BMI scores were found to be positively correlated with the SOFA, APACHE II, and NUTRIC scores, while an inverse relationship was observed with CRP, PCT, and IL-6 levels. This inverse correlation implies that an enhanced nutritional status may correlate with reduced inflammatory activity. Within the subgroup of sepsis patients with sarcopenia, these associations were even more pronounced, indicating that the presence of malnutrition and sarcopenia could be linked to heightened inflammation and a greater risk of organ dysfunction.

The findings from these analyses underscore the intricate relationship between a patient’s nutritional status and systemic inflammation, and they highlight the significance of considering both nutritional and inflammatory parameters in the comprehensive care of sepsis patients. This holistic approach may facilitate more effective treatment strategies and improved patient outcomes.

**Figure 6:**
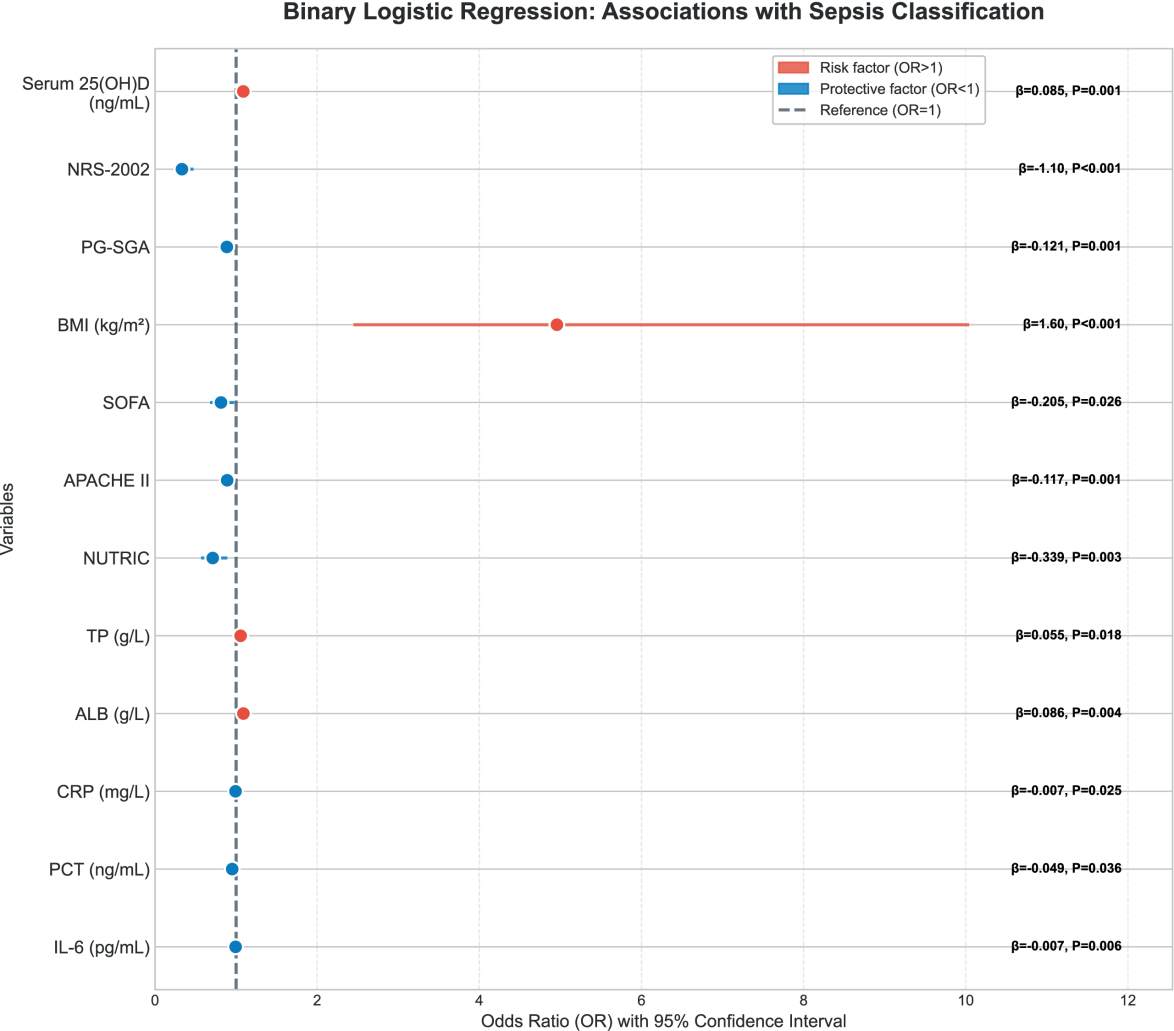
Binary logistic regression model addressing the associations between Serum 25(OH)D, nutrition indicators, infection markers and sepsis classification.

In the binary logistic regression analysis, the sepsis cohort was bifurcated into two distinct groups based on the presence or absence of sarcopenia, with these groups serving as the dependent variables. The outcomes of this analysis are delineated in Figure 6, The detailed underlying data are provided in Table 6. Serum 25(OH)D levels were significantly associated with sepsis classification in the binary logistic regression model (*β* = 0.085, OR = 1.089, 95% CI: 1.034–1.147, *p* = 0.001). The OR was calculated to be 1.089, with a 95% CI ranging from 1.034 to 1.147, indicating that for every unit increase in serum 25(OH)D, there is an 8.9% increase in the odds of a favorable outcome.

**Table 6:** Binary logistic regression model addressing the associations between serum 25(OH)D, nutrition indicators, infection markers and sepsis classification.

Characteristic	*β*	*p*	OR	CI (95%)
Serum 25(OH)D (ng/mL)	0.085	0.001	1.089	1.034–1.147
NRS-2002	−1.103	<0.001	0.332	0.233–0.474
PG-SGA	−0.121	0.001	0.886	0.826–0.950
BMI (kg/m^2^)	1.601	<0.001	4.957	2.448–10.040
SOFA	−0.205	0.026	0.814	0.680–0.975
APACHE II	−0.117	0.001	0.890	0.833–0.951
NUTRIC	−0.339	0.003	0.712	0.569–0.892
TP (g/L)	0.055	0.018	1.056	1.009–1.105
ALB (g/L)	0.086	0.004	1.090	1.027–1.157
CRP (mg/L)	−0.007	0.025	0.993	0.987–0.999
PCT (ng/mL)	−0.049	0.036	0.952	0.909–0.997
IL-6 (pg/mL)	−0.007	0.006	0.994	0.989–0.998

NRS-2002, Nutrition Risk Screening 2002; PG-SGA score, Patient-Generated Subjective Global Assessment score; BMI, Body Mass Index; SOFA, Sequential Organ Failure Assessment; APACHE II, Acute Physiology and Chronic Health Evaluation II; NUTRIC, Nutrition Risk in Critically Ill; TP, Total Protein; ALB, Albumin; CRP, C-reactive protein; PCT, Procalcitonin; IL-6, Interleukin 6.

Furthermore, nutritional indicators demonstrated a negative correlation with the severity of inflammation, with this association reaching statistical significance (*p* < 0.05). This suggests that improved nutritional status is inversely related to inflammatory severity, reinforcing the potential mitigating role of adequate nutrition in modulating the inflammatory response in sepsis.

**Figure 7:**
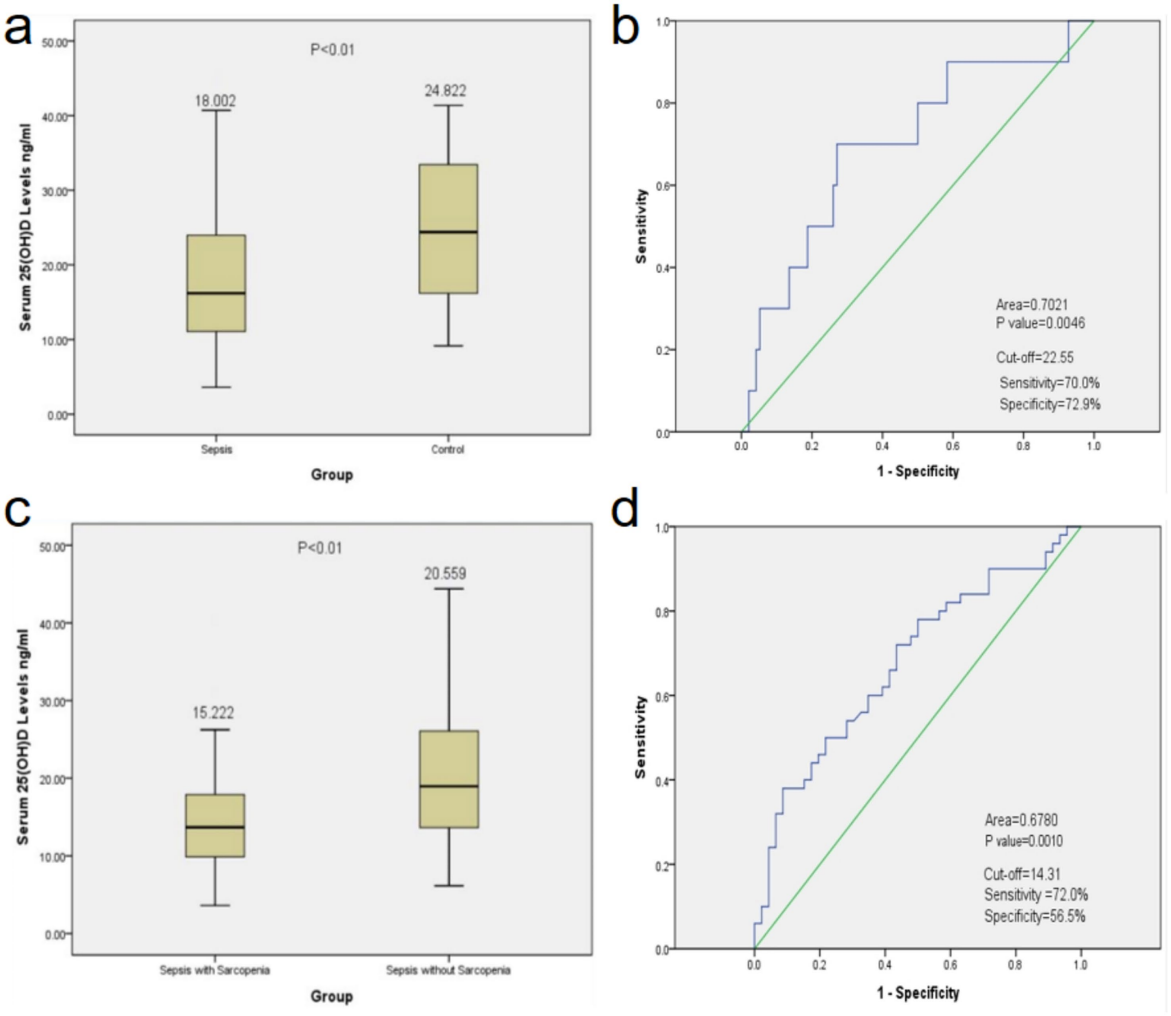
Analysis of 25(OH)D cut-off values for diagnosing Sepsis. Means and *p*-values of serum 25(OH)D levels in the sepsis and control groups **(a)**, and the sepsis with Sarcopenia and without Sarcopenia groups **(c)**. The ROC analysis to determine optimal cut-off values in the sepsis and control groups **(b)**, and the sepsis with sarcopenia and without sarcopenia groups **(d)**. ROC, receiver operating characteristic.

The ROC curve analyses, along with the determination of optimal cut-off values, are illustrated in Figure 7. Notably, significant differences in serum 25(OH)D levels were observed when comparing the sepsis and control groups, as well as between the sepsis with sarcopenia and the sepsis without sarcopenia groups (as depicted in Figures 7a,c). For the sepsis versus control group comparison, the AUC was calculated to be 0.7021, indicating that 25(OH)D demonstrated a sensitivity of 70.0% and a specificity of 72.9%, indicating a moderate discriminatory performance. In contrast, the AUC for distinguishing the sepsis-combined-sarcopenia group from the non-sarcopenia group was 0.6780, with a sensitivity of 72.0% and a specificity of 56.5%, suggesting a modest discriminatory value.

The optimal cut-off values for 25(OH)D, showing the best discriminatory performance for distinguishing sepsis and sepsis with sarcopenia, were identified as 22.55 ng/mL and 14.31 ng/mL, respectively (as shown in Figures 7b,d). These cut-off values may serve as potential discriminatory reference points within the present study population, but should not be interpreted as universal diagnostic thresholds. Consequently, in clinical settings, a serum 25(OH)D level below 22.55 ng/mL may be employed as an indicator for diagnosing sepsis. Furthermore, when the 25(OH)D level is below 14.31 ng/mL, in conjunction with other diagnostic criteria, it may be inferred that a patient with sepsis is also suffering from sarcopenia. These findings underscore the utility of 25(OH)D as a potential biomarker reflecting the nutritional and inflammatory status of patients with sepsis, particularly those with sarcopenia.

## 4 Discussion

In the current investigation, a high prevalence of severe malnutrition and inflammatory conditions was noted among patients with sepsis, corroborating previous studies (31–33). The ubiquity of malnutrition in this patient population is underscored by diminished BMI and reduced levels of TP and ALB (34–36). Concurrently, pronounced inflammatory responses are indicated by increased serum concentrations of CRP, PCT and IL-6 (37–41). These observations are in line with existing literature that recognizes inflammation and malnutrition as prevalent clinical manifestations in sepsis, suggesting their integral roles in the pathophysiology of the condition and their potential influence on the evolution and severity of sepsis (42, 43).

This study further revealed that the sepsis cohort with comorbid sarcopenia exhibited exacerbated malnutrition and more adverse clinical outcomes. Patients in this subgroup were characterized by an elevated mean age, higher nutritional scores as measured by the NRS-2002 and PG-SGA, and a decreased BMI. These findings imply a close relationship between malnutrition and the development and exacerbation of sarcopenia in sepsis patients. Prior research has also established a higher incidence of malnutrition in sarcopenic patients, with nutritional status being intricately linked to sarcopenia severity (11, 44). These findings underscore the importance of nutritional and muscle-related assessments in patients with sepsis, particularly in those with sarcopenia. However, given the cross-sectional design of the present study, the observed associations should not be interpreted as evidence of causality or as proof of intervention efficacy.

Serum 25(OH)D levels were identified as a significant parameter in this study, with sepsis patients showing significantly lower levels compared to controls. A negative correlation was observed between 25(OH)D levels and the onset and progression of sepsis. Notably, the subgroup of patients with sepsis and myasthenia gravis presented with even lower 25(OH)D levels, aligning with previous findings that have reported reduced levels in sepsis (7, 45–47) and myasthenia gravis (10, 48–50) patients. These data hint at a pivotal role for 25(OH)D in the pathogenesis of sepsis, with its deficiency potentially contributing to malnutrition and sarcopenia in affected patients.

Nutritional support remains an important component of sepsis care, and previous studies have explored its possible relationship with clinical outcomes (51–55). However, the present study did not evaluate the effects of vitamin D supplementation or nutritional intervention. Therefore, our findings should be interpreted as observational associations only, and the potential benefits of these interventions in patients with sepsis and sarcopenia require confirmation in prospective interventional studies.

This study has several limitations. First, it was conducted at a single center, and the study population was derived from one hospital, which may limit the generalizability of the findings to other regions, healthcare settings, or patient populations. Second, the sample size was relatively limited, particularly for subgroup analyses, which may reduce the robustness of the statistical analyses and increase the possibility of random error or type II error. Third, because this was a cross-sectional study, the observed associations between serum 25(OH)D levels and nutritional or inflammatory indicators should be interpreted as correlations rather than causal relationships. In addition, potential confounding factors affecting serum 25(OH)D levels, such as sunlight exposure, previous vitamin D supplementation, seasonal variation, and metabolic comorbidities, were not systematically collected or controlled for in the present study. Finally, dynamic changes in vitamin D status and the effects of interventions were not evaluated. Therefore, larger multicenter prospective studies are needed to validate the present findings and further clarify the clinical relevance of vitamin D in patients with sepsis and sarcopenia.

## 5 Conclusion

Malnutrition and inflammation were common in patients with sepsis and appeared to be more pronounced in those with concomitant sarcopenia. Serum 25(OH)D levels were associated with nutritional status and inflammatory markers in patients with sepsis, particularly in those with sarcopenia, suggesting that 25(OH)D may serve as a potential biomarker reflecting these clinical features. However, because of the single-center cross-sectional design and limited sample size, these findings should be interpreted with caution. Larger multicenter prospective studies are needed to validate these associations and to further determine the clinical relevance of vitamin D in this population.

## Data Availability

The original contributions presented in the study are included in the article/supplementary material, further inquiries can be directed to the corresponding author.

## Footnotes

1
http://www.chictr.org.cn/index.aspx
